# Psychological Distress in Bladder Cancer Patients: A Systematic Review

**DOI:** 10.1002/cam4.70345

**Published:** 2024-11-13

**Authors:** Kezia Reji Thomas, Catherine Joshua, Christine Ibilibor

**Affiliations:** ^1^ University of Virginia Charlottesville Virginia USA

**Keywords:** anxiety, bladder cancer, depression, oncology, psychological distress

## Abstract

**Objective:**

Bladder cancer patients experience high levels of disease and treatment‐related distress, however, factors that can mitigate patient‐reported psychological distress are poorly characterized. Thus, this study serves to summarize the burden of psychological distress among bladder cancer patients and identify clinical, psychological, and socioeconomic factors that are associated with varying levels of psychological distress.

**Methods:**

We performed a systematic review of studies examining psychological distress in bladder cancer patients. We searched PubMed/MEDLINE, Embase, and PsycINFO from October 2000 to February 2024 according to the PRISMA guidelines. Associations between clinical, psychological, socioeconomic factors, and psychological distress were identified in each study and extracted. The protocol for this review is registered in PROSPERO (CRD42024495568).

**Results:**

Using our search strategy, 759 articles were retrieved and 17 met inclusion criteria, representing 2572 bladder cancer patients. Tumor stage (*n* = 3), younger age (*n* = 2), female sex (*n* = 2) the preoperative setting (*n* = 2), depression/anxiety (*n* = 2), and negative psychological response to illness (*n* = 2) were common factors associated with increased psychological distress. Transitioning from the preoperative to the postoperative period (*n* = 2), postoperative inpatient rehabilitation (*n* = 2), feeling well informed (*n* = 2), and social support (*n* = 2) were associated with decreased psychological distress.

**Conclusion:**

While clinical factors associated with increased psychological distress are nonmodifiable, clinical, psychological, and socioeconomic factors associated with decreased psychological distress can be improved upon by healthcare providers to mitigate the distress that bladder cancer patients experience.

## Graphical abstract

This systematic review summarizes the burden of psychological distress among bladder cancer patients and identifies associated clinical, psychological, and socioeconomic factors. We found that advanced tumor stage, younger age, female gender, preoperative settings, depression/anxiety, and negative psychological responses increase distress, while transitioning to postoperative care, postoperative rehabilitation, feeling well‐informed, and social support mitigate it.

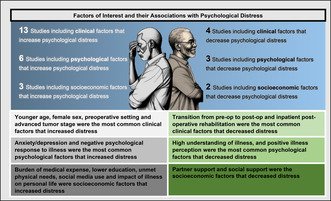

## 1 Introduction

Bladder cancer stands as one of the most prevalent urological malignancies globally, totaling over 573,278 cases and 212,536 deaths in the year 2020 alone [1]. Projections indicate a disconcerting upward trend. In 2024, the American Cancer Society (ACS) projected that 83,190 individuals would be diagnosed with bladder cancer, and 16,840 of them would die from the disease. This represents an increase of approximately 1000 new cases, and over 130 more fatalities compared to the previous year's predictions [2, 3]. Moreover, there is an estimated 73% increase in annual cases and an 87% surge in deaths predicted by 2040 on a global scale [4].

Notably, bladder cancer patients undergoing treatment such as neoadjuvant chemotherapy, radical cystectomy (RC), urinary diversion, and urinary reconstruction experience changes in their physical and mental health‐related quality of life as a consequence of these therapies [5]. Fecal urgency, loss of sexual and urinary function, fecal leakage, and recurrent urinary tract infections are a few physical side effects of bladder cancer treatment [6, 7, 8]. Moreover, a bladder cancer recurrence either locally or distantly can further increase the level of treatment‐related burden that bladder cancer patients experience [9].

While considerable attention has been devoted to understanding the physical ramifications of major surgical procedures, and local and systemic therapies in bladder cancer patients, there has been less robust investigation of their psychological impact, despite the significant amount of psychological distress bladder cancer patients shoulder relative to the general population [10, 11]. As a result, the investigation of psychological distress in bladder cancer patients remains a growing area of study [11, 12]. The consequences of psychological distress have both quality‐of‐life and clinical implications including anxiety, depression, suicidality, and decreased social functioning [13, 14]. Various factors have been found to decrease cancer treatment related psychological distress such as psychosocial support and resilience in other cancers such as breast and prostate [15, 16]. However, few studies have examined predictors or factors like positive psychological adaptation that can decrease psychological distress in bladder cancer patients [12]. Thus, the objective of this review is to present a comprehensive overview of existing studies examining psychological distress in bladder cancer patients and to identify the impact of clinical and psychological factors on this distress. This review aims to answer the following research questions:

- What is the prevalence of psychological distress in bladder cancer patients?
- What is the impact of given clinical, psychological, and socioeconomic factors on psychological distress in bladder cancer patients?

## 2 Materials and Methods

### 2.1 Study Design

This review was conducted according to the Preferred Reporting Items for Systematic Reviews and Meta‐Analysis (PRISMA) guidelines [17]. The protocol for this review was published in the International Prospective Register of Systematic Reviews (PROSPERO, CRD42024495568).

### 2.2 Selection Criteria

Studies were included if they fulfilled the following inclusion criteria: published between October 2000 and February 2024, in a peer‐reviewed journal, published in English or had an English translation available, included bladder cancer patients with any tumor stage and at any stage of treatment, provided either quantitative or qualitative data on psychological distress, and discussed the presence of a positive or negative association with a clinical, psychological or socioeconomic factor, and psychological distress. Studies with only nonbladder cancer patients, those without a measure of psychological distress, and those without a discussion of an association between a given factor, clinical, psychological or socioeconomic, and psychological distress were excluded.

### 2.3 Database Search

To capture the breadth of data present on psychological distress in bladder cancer patients, we searched PubMed/MEDLINE, Embase, and PsycINFO and included all study design and publication types. In collaboration with a medical librarian (CJ), the following terms were identified and combined to find eligible articles: “bladder cancer” OR “urinary bladder neoplasms” OR “urologic neoplasms” AND “psychological stress” OR “psychological distress” OR “emotional stress” OR “emotional distress” OR “cognitive stress” OR “cognitive distress.” Where possible, our keyword terms were translated into the terminologies of their respective databases. Gray literature was not searched given our inclusion and exclusion criteria (see the Supporting Information for the full search strategy).

### 2.4 Study Selection

A two round screening approach was used. Articles that were identified using our search strategies were first screened independently by two authors (KRT and CI), using only the article title and abstract. We used a piloted checklist to identify articles meeting inclusion criteria, and then screened the full text. In cases of disagreement, consensus was reached through discussion.

### 2.5 Data Extraction, Data Synthesis, and Quality Assessment

A piloted data abstraction tool was used by two authors (KRT and CI) to extract data from each included article, independently. We collected article characteristics such as year and country of publication, sample size, follow‐up duration, primary study outcome, study design, and study type. We collected data on study population clinical characteristics such as sex, age, distribution of bladder cancer stage, surgical management, and use of chemotherapy. To determine the extent of psychological distress among bladder cancer patients in each study, we collected data regarding the type of surveys used to measure psychological distress, survey scores, and the prevalence of psychological distress expressed as a percentage when present. To examine the effect of clinical, psychological, and socioeconomic factors on psychological distress, we collected descriptive data on their negative or positive association with psychological distress. Descriptions of the association between given factors and psychological distress were coded iteratively by two authors (KRT and CI) until each given factor was assigned a coded title.

Study quality was assessed using the Study Quality Assessment Tools of the National Institute for Health and implemented specific tools based on study type [18]. Each study was given a quality score of poor, fair, or good.

## 3 Results

### 3.1 Study Selection, Study Design Types, Characteristics and Quality

**FIGURE 1:**
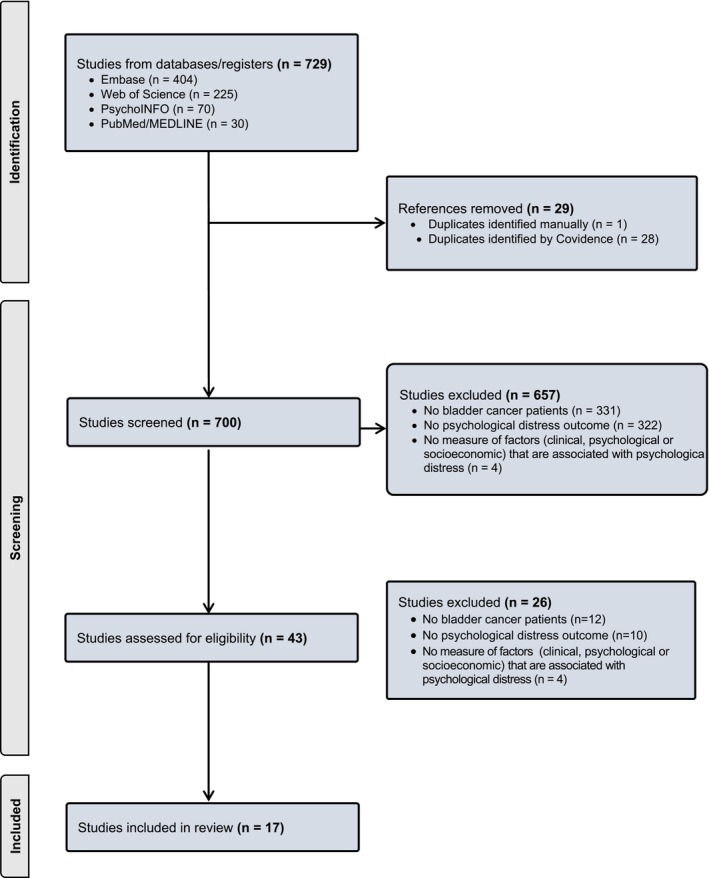
PRISMA diagram.

**FIGURE 2:**
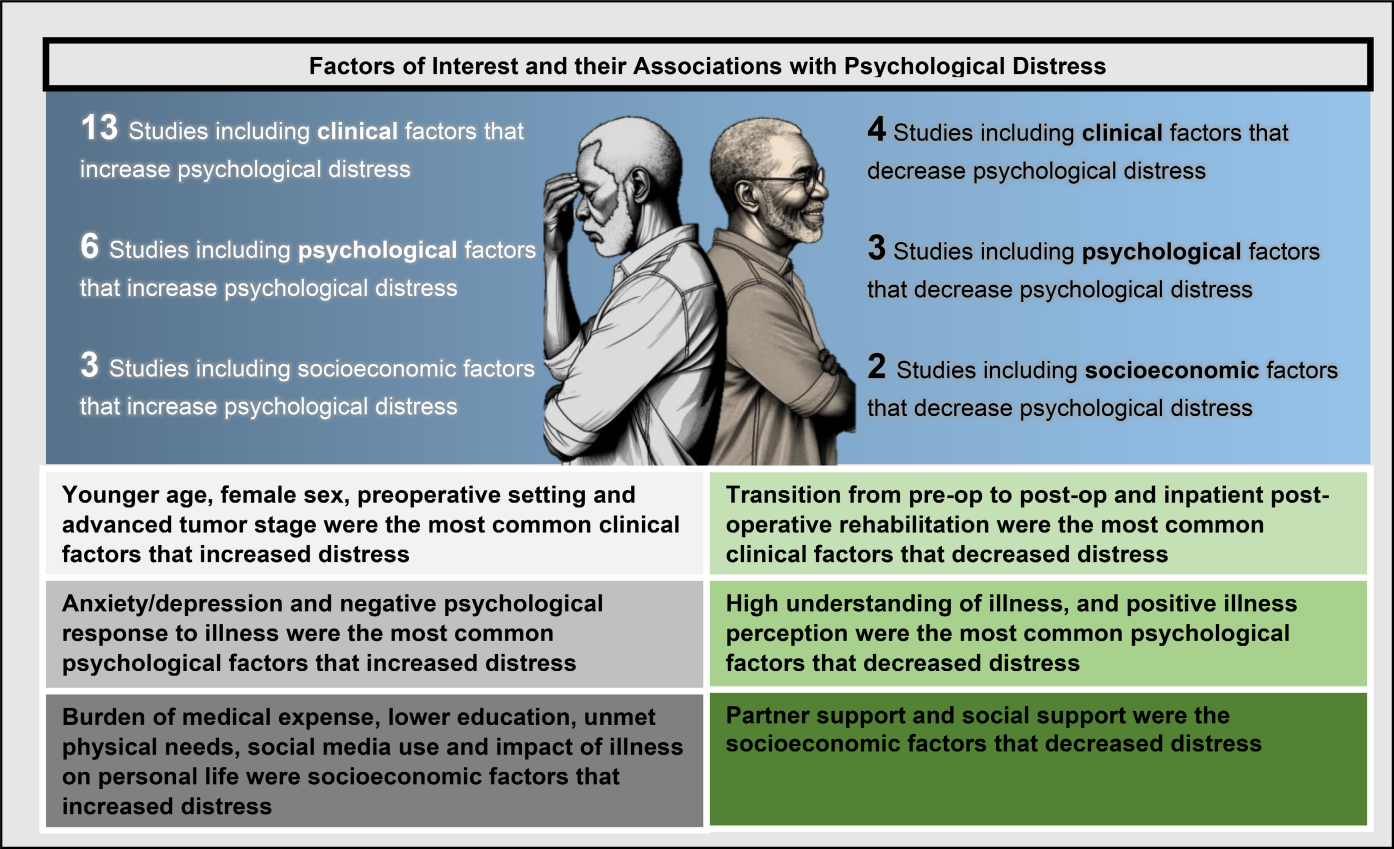
Clinical, psychological, and socioeconomic factors associated with psychological distress in bladder cancer.

Using our search strategy, 759 articles were retrieved. After removing duplicates, 700 articles were screened by title and abstract. The remaining 43 articles were read in full by two authors (KRT and CI) and 17 articles met inclusion criteria (Figures 1 and 2) [19, 20, 21, 22, 23, 24, 25, 26, 27, 28, 29, 30, 31, 32, 33, 34, 35]. A common reason for article exclusion after the full‐text review was lack of evaluable bladder cancer patients (*n* = 12) which precluded an analysis of the level of psychological distress specific to that population. Other reasons for exclusion included lack of a psychological distress outcome to examine (*n* = 10) and lack of a demonstrable association between a given factor and psychological distress (*n* = 4). Most studies were cross‐sectional studies (*n* = 9) or prospective cohort studies (*n* = 5) in study design followed by retrospective (*n* = 2) and case–control studies (*n* = 1). Most studies were conducted in the United States (*n* = 4) and Germany (*n* = 4) followed by China (*n* = 3), Australia (*n* = 2), Canada (*n* = 2), Austria (*n* = 1), and Spain (*n* = 1). Many studies evaluated patients in the postoperative setting (*n* = 6) or pre‐ and postoperatively (*n* = 4). All studies were given a study quality score of good as each study had a clearly stated objective, study population, exposure measures, outcome measures, and inclusion and exclusion criteria.

### 3.2 Prevalence of Psychological Distress Among Bladder Cancer Patients

The 17 included studies represent 2572 bladder cancer patients, of which approximately 485 were women. Sample sizes ranged from *n* = 54 to *n* = 327 and the mean age of included patients ranged from 71 to 62. Across the included studies that reported psychological distress prevalence data, the percentage of patients who were categorized as having psychological distress ranged from 78% to 3.6% (mean: 32.3%, 95% CI: 11.3%–42.5%) (Tables 1, 2, 3). The most commonly used validated questionnaires administered to quantify psychological distress in bladder cancer patients was the Hospital Anxiety and Depression Scale (HADS) (*n* = 6) followed by the Distress Thermometer (DT) (*n* = 3), the Kessler‐10 (*n* = 1), the Basic Symptom Inventory‐18 (*n* = 1), the Brief Symptom Index (*n* = 1), the Edmonton Symptom Assessment System revised (*n* = 1), and the Perceived Stress Scale‐10 (*n* = 1). Two studies utilized nonvalidated questionnaires. Common cut‐off values for these questionnaires to mark the presence of psychological distress were ≥ 8 for the HADS and > 4 for the DT.

**TABLE 1:** Association between clinical factors and psychological distress in bladder cancer patients.

Study	Country	Study design	Primary study objective	Patients (n)	Questionnaires	Prevalence (n, %)	Setting	Factor	Description of the association
**Studies examining clinical factors that increased psychological distress**									
Ajaj et al. [20]	Canada	Cross‐sectional study	Assess age‐based differences in psychological and physical symptoms of bladder cancer patients at different disease stages.	232	Edmonton Symptom Assessment System‐revised (ESAS‐r)	Not reported	Presurgical, postsurgical	Younger age	Patients younger than 65 compared to patients over 65 had higher psychological distress sub‐score (PDSS) within the ESAS‐r at diagnosis, and after RC (*p* = 0.023)
								Number of prior TURBTs	Higher number of prior TURBTs was associated with higher distress (*β* = 1.602, *p* = 0.017)
Draeger et al. [21]	Germany	Cross‐sectional study	Screen for the presence of distress among bladder cancer patients stratified by clinicopathologic factors	301	National Comprehensive Cancer Network (NCCN) Distress Thermometer (NCCN‐DT)	157, 52.2%	Presurgical, postsurgical, prechemo, postchemo	Chemotherapy	Patients on chemotherapy had higher mean distress scores (mean NCCN DT 5.7, *p* < 0.001) compared to the total group (mean NCCN DT 4.6)
								Advanced pathological/ tumor stage	Patients with progressive disease had higher mean (mean NCCN DT 5.4, *p* < 0.001) compared to the total group (mean NCCN DT 4.6)
Heyes et al. [22]	Australia	Prospective cohort	Explore the associations between distress and both the functional impact of bladder cancer and its perceived psychological burden	119	The Mini‐mental Adjustment to Cancer	Not reported	Post‐surgical	Illness duration	Longer illness duration in years (*β* = 0.24, *p* = 0.01) was associated with higher levels of distress
Joshy et al. [23]	Australia	Case–control study	Quantify short‐ and long‐term physical and mental health outcomes in people with and without cancer	398	Kessler‐10 (K10)	60, 19.7%	Not reported	Recent treatment for cancer	28.6% (19/56) of bladder cancer patients treated within the last 1 month reported moderate to high distress
Kowalkowski et al. [24]	United States	Cross‐sectional study	Examine the association between current smoking status, and adherence to surveillance guidelines among bladder cancer patients	109	Brief Symptom Index (BSI)	Not reported	Pre‐surgical	Smoking use while getting surveillance cystoscopy	Mean distress scores for current smokers undergoing surveillance cystoscopy were higher than non‐smokers undergoing surveillance (10.6 vs. 5.8, *p* = 0.03)
Mohamed et al. [27]	United States	Cross‐sectional study	Assess the prevalence of clinical predictors for psychological distress among bladder cancer patients	159	Hospital Anxiety and Depression Scale (HADS)	Not reported	Post‐surgical	Younger age	Psychological distress was significantly associated with younger age (*p* < 0.05)
Palapattu et al. [28]	United States	Prospective cohort	Determine the prevalence of psychological distress in bladder cancer patients before and after a radical cystectomy	74	Basic Symptom Inventory‐18 (BSI)	33, 45% preoperatively	Pre‐surgical; Post‐surgical	Pre‐operative setting	Mean BSI scores among radical cystectomy patients were elevated preoperatively (11.4, SD = 10.8)
								Advanced pathological/ tumor stage	High (T3 or T4) compared to low (T1 or T2) pathological tumor stage was associated with high postoperative distress (odds ratio: 3.9, 95% CI 1.05 to 14.2, *p* = 0.042)
Pastore et al. [29]	Spain	Cross‐ sectional study	Evaluate psychological distress preoperatively in urologic cancer patients undergoing radical cystectomy, radical prostatectomy, radical nephrectomy, or transurethral resection	125	Hospital Anxiety and Depression Scale (HADS, psychological distress = 8–10 on anxiety and depression subscales) and State–Trait Anxiety Inventory (STAI).	Anxiety: 19, 9.8%, Depression: 7, 3.6%	Pre‐surgical	Radical cystectomy	Radical cystectomy patients had higher mean distress scores (STAI‐State 23.93, *p* = 0.032) compared to patients undergoing nephrectomy (22.75), prostatectomy (18.16), and TURBT (18.12)
								Female gender	Female patients had higher mean distress scores compared to males (STAI‐Trait, 22.65 vs. 16.83, *p* = 0.038)
Qian et al. [30]	China	Prospective cohort	Evaluate the influence of depressive and anxiety symptoms on the 1‐year recurrence rate in non‐muscle invasive bladder cancer patients.	104	Hospital Anxiety and Depression Scale (HADS)	Anxiety: 28%, Depression: 33%	Postsurgical	Chronic pain	Chronic pain was associated with higher having anxiety symptoms (odds ratio: 3.447, *p* = 0.023)
								Preoperative bladder irritation	Preoperative bladder irritation prior to TURBT was associated with higher depressive symptoms (odds ratio: 9.57, *p* = 0.002) and higher anxiety symptoms (odds ratio: 6.89, *p* = 0.009)
								Advanced pathological/tumor stage	Clinical stage T1 compared to Ta among NMIBC patients (OR = 7.4498, *p* = 0.006) was associated with psychological distress and depressive symptoms
Ralston et al. [31]	United States	Retrospective cohort	Identify prevalence of distress, associated clinical risk factors and psychosocial referral trends in the bladder cancer population	81	Distress Thermometer (DT, DT > 4) and Patient Health Questionnaire‐9 (PHQ‐9)	11, 13.6%	Not reported	Closeness to death/within 6 months of death	54% of bladder cancer patients had elevated distress within 6 months of their death
								Metastatic disease	45% of bladder cancer patients with elevated distress had metastatic disease
								Female sex	36% (4/11) of patients with elevated distress were female, and the female sex was associated with elevated distress
Trudel et al. [34]	Canada	Cross‐sectional study	Evaluate the health‐related quality of life in radical cystectomy patients before and after surgery	54	Distress Thermometer (DT)	Not reported	Presurgical, postsurgical	Preoperative setting	Median DT scores were higher preoperatively
**Studies examining clinical factors that decreased psychological distress**									
Licht et al. [25]	Austria	Cross‐sectional study	Analyze the impact of cancer rehabilitation on health‐related quality of life, psychological distress, and somatic symptoms of cancer survivors.	99	Hospital Anxiety and Depression Scale (HADS)	Not reported	Postsurgical	In‐patient postoperative rehabilitation	A 21‐day multidisciplinary rehabilitation resulted in a decrease in mean depression (6.4–4.2, *p* < 0.001) and anxiety scores (6 to 4.7, *p* < 0.001) in bladder cancer patients
Palapattu et al. [28]	United States	Prospective cohort	Determine the prevalence of psychological distress in bladder cancer patients before and after a radical cystectomy	74	Basic Symptom Inventory‐18 (BSI)	21, 34% 1 month postoperatively	Presurgical, postsurgical	Transition from the pre‐ to postoperative setting	Mean distress scores were lower postoperatively compared to preoperative scores (9.3 vs. 11.4, *p* = 0.028)
Schulz et al. [32]	Germany	Cross‐sectional study	Assess patients' needs after radical cystectomy and investigate the efficacy of in‐patient early rehabilitation to improve functional outcomes and prevent postoperative complications.	103	Nonvalidated survey	46, 44.7%	Postsurgical	In‐patient postoperative rehabilitation	30.8% of the patients indicated an improvement in their psychological distress after in‐patient rehabilitation
Trudel et al. [34]	Canada	Cross‐sectional study	Evaluate the health‐related quality of life in radical cystectomy patients before and after surgery	54	Distress Thermometer (DT)	Not reported	Presurgical, postsurgical	Transition from the pre‐ to postoperative setting	Distress scores were higher preoperatively compared to postoperatively (*p* = 0.005)

### 3.3 Clinical Factors That Increased or Decreased Psychological Distress

Among the included articles, 13 reported clinical factors that were associated with increased psychological distress (Table 1) [20, 21, 22, 23, 24, 25, 27, 28, 29, 30, 31, 32, 34]. The most common clinical factors that were associated with increased psychological distress in bladder cancer patients were younger age (*n* = 2), female sex (*n* = 2), advanced pathological/tumor stage (*n* = 2), and the preoperative setting (*n* = 2) [20, 21, 27, 28, 29, 31]. One study reported that patients younger than 65 had higher distress scores compared to patients above this age [20]. Another study noted that women reported higher distress scores compared to men (State‐Trait Anxiety Inventory‐Trait, 22.65 vs. 16.83, *p* = 0.038), while another reported that female sex was associated with higher levels of distress [29, 31]. Patients with progression of their cancer reported higher psychological distress on the DT (5.4 vs. 4.6, *p* < 0.001) compared to the total group in one study, whereas another study reported a higher likelihood of elevated psychological distress in patients with high versus low pathologic tumor stage (odds ratio: 3.9, 95% CI 1.05 to 14.2, *p* = 0.042) [21, 28]. Patients undergoing surgery reported higher distress scores in the preoperative setting compared to the postoperative setting [28, 34].

Of the included studies, four reported clinical factors that were associated with decreased psychological distress (Table 1) [25, 28, 32, 34]. The most common clinical factors that were associated with decreased psychological distress in bladder cancer patients were inpatient postoperative rehabilitation (*n* = 2) and transitioning from the pre‐ to postoperative setting (*n* = 2) [25, 28, 32, 34]. One study reported that 30.8% of patients who completed inpatient rehabilitation postoperatively experienced a reduction in their psychological distress [32]. Similarly, another study reported a decrease in mean depression (6.4–4.2, *p* < 0.001) and anxiety scores (6–4.7, *p* < 0.001) on the HADS after inpatient rehabilitation while two studies reported a decrease in distress scores in patients as they moved from the preoperative to the postoperative period [25, 28, 34].

### 3.4 Psychological Factors That Increased and Decreased Psychological Distress

Among the included articles, six reported psychological factors that were associated with increased psychological distress (Table 2) [19, 22, 26, 30, 33, 35]. The most common psychological factors that were associated with increased psychological distress in bladder cancer patients were negative psychological response to illness (*n* = 2) and depressive/anxiety symptoms (*n* = 2) [19, 22, 26, 35]. The perception of a high psychological burden of their cancer quantified as lower scores on the Bladder Cancer Index survey among bladder cancer patients was associated with higher distress scores on the Mini‐mental Adjustment (*β* = −0.42, *p* ≤ 0.001) [22]. In addition, a negative emotional response was positively correlated with distress quantified by the anxiety (*ρ* = 0.860, *p* < 0.001) and depressive (*ρ* = 0.844, *p* < 0.001) symptoms domains of the HADS [35]. Depressive and anxiety symptoms on the HADS were positively correlated with psychological distress measured by the Perceived Stress Scale‐10 and Questionnaire on Stress in Cancer Patients (QSC; *Fragebogen zur Belastung von Krebskranken*, FBK‐R23) (depressive: *ρ* = 0.708, and anxiety: *ρ* = 0.733, *p* < 0.001) [19, 26].

**TABLE 2:** Association between psychological factors and psychological distress in bladder cancer patients.

Study	Country	Study design	Primary study objective	Patients (*n*)	Questionnaires	Prevalence (*n*, %)	Setting	Factor	Description of the association
**Studies examining psychological factors that increase psychological distress**									
Heyes et al. [22]	Australia	Prospective cohort	Explore the associations between distress, the functional impact of bladder cancer and its perceived psychological burden	119	The Mini‐Mental Adjustment to Cancer	Not reported	Postsurgical	Negative psychological response to illness	Poorer perceived psychological burden scores (*β* = −0.42), and poorer evaluation of bladder/sexual/bowel function (*β* = −0.31) were related to higher levels of distress
Li et al. [19]	China	Cross‐sectional study	Explore the association between psychological stress, depressive and anxiety symptoms among bladder and renal cancer patients	327	Perceived Stress Scale‐10 (PSS‐10)	Anxiety: 71.3%, Depression: 78.0%	Postsurgical	Depressive/anxiety symptoms	Depressive and anxiety symptoms were positively related to psychological distress
Mani et al. [26]	Germany	Retrospective cohort	Evaluate the prevalence of mental distress in patients with newly diagnosed bladder cancer	101	HADS and the Fragebogen zur Belastung von Krebskranken (FBK‐R23)	Anxiety: 25.3%, Depression: 25.3%, FBK‐R23: 21.4%	Presurgical	Depressive/anxiety symptoms	Depression (*ρ* = 0.708, *p* < 0.001) and anxiety (*ρ* = 0.733, *p* < 0.001) positively correlated with distress
Qian et al. [30]	China	Prospective cohort	Evaluate the influence of anxiety and depressive symptoms on the 1‐year recurrence rate in nonmuscle invasive bladder cancer patients	104	HADS	Anxiety: 28%, Depression: 33%	Postsurgical	Less understanding of the disease	Less understanding of the disease was associated with anxiety, and depressive symptoms (odds ratio = 4.357, *p* = 0.037)
Volz et al. [33]	Germany	Prospective cohort	Assess predictive factors for high fear of cancer recurrence in patients undergoing surgery for genitourinary cancer	85	11‐Item Likert Scale	Not reported	Presurgical and Postsurgical	Fear of cancer recurrence	High preoperative distress was associated with higher fear of cancer recurrence scores before (median 20, *p* = 0.001) and after surgery (median 14, *p* = 0.017)
Zhang et al. [35]	China	Prospective cohort study	Explore the value of illness perceptions in predicting psychological distress	101	HADS	Anxiety: 12.8% and 5%, at 3 and 12 months. Depression: 6.9% and 16.8%, at 3 and 12 months	Postsurgical	Perceived personal impact of illness	Negative Illness Perceptions item consequences (impact of illness on person's life) were positively correlated with anxiety (*ρ* = 0.854 and 0.853, *p* < 0.001) and depressive (*ρ* = 0.842 and 0.837) symptoms at the 3 and 12 months of follow‐up
								Perceived illness duration	Negative illness perceptions item timeline (perceived illness duration) was positively correlated with anxiety and depressive symptoms at the 3‐ and 12‐month follow‐up
								Personal experience of illness symptoms	Negative Illness Perceptions item identity (experienced symptoms of illness) was positively correlated with anxiety (*ρ* = 0.880 and 0.863, *p* < 0.001) and depressive (*ρ* = 0.794 and 0.813) symptoms at the 3 and 12 months of follow‐up
								Personal concern about illness	Negative Illness Perceptions item concern (the extent of concern about illness) was positively correlated with anxiety (*ρ* = 0.465 and 0.430, *p* < 0.001) and depressive (*ρ* = 0.484 and 0.495) symptoms at the 3 and 12 months of follow‐up
								Negative psychological response to illness	Negative Illness Perceptions item emotional representation (emotional response to illness) was positively correlated with anxiety (*ρ* = 0.860 and 0.865, *p* < 0.001) and depressive (*ρ* = 0.844 and 0.814) symptoms at the 3 and 12 months of follow‐up
**Studies examining psychological factors that decreased psychological distress**									
Li et al. [19]	China	Cross‐sectional study	Explore the association between psychological stress, depressive and anxiety symptoms among bladder and renal cancer patients	327	Perceived Stress Scale‐10 (PSS‐10)	Anxiety: 71.3%, Depression: 78.0%	Postsurgical	Resilience	Resilience was negatively associated with anxiety (*ρ* = −0.499, *p* < 0.01), depressive symptoms (*ρ* = −0.509, *p* < 0.01), and psychological distress (i = −0.437, *p* < 0.01)
Mani et al. [26]	Germany	Retrospective cohort	Evaluate the prevalence of mental distress in patients with newly diagnosed bladder cancer	101	HADS and the Fragebogen zur Belastung von Krebskranken (FBK‐R23)	Anxiety: 25.3%, Depression: 25.3%, FBK‐R23: 21.4%	Presurgical	Feeling well informed	Feeling well informed was negatively correlated with FBK‐R23 scores (*i* = −0.335, *p* < 0.001), anxiety (*ρ* = −0.323, *p* < 0.001) and depressive (*ρ* = −0.332, *p* < 0.001) symptoms
Zhang et al. [35]	China	Prospective cohort study	Explore the value of illness perceptions in predicting psychological distress	101	HADS	Anxiety: 12.8% and 5%, at 3 and 12 months. Depression: 6.9% and 16.8% at 3 and 12 months	Postsurgical	Feeling well informed	Positive Illness Perceptions item understanding was negatively correlated with anxiety (*ρ* = −0.786 and − 0.725, *p* < 0.001) and depressive symptoms (*ρ* = −0.669 and −0.647) at the 3 and 12 months of follow‐up
								Belief in own's personal control over the illness	Positive Illness Perceptions item personal control (the individual beliefs of control over the illness) negatively correlated with anxiety (*ρ* = −0.355 and −0.416, *p* < 0.001) and depressive symptoms (*ρ* = −0.448 and −0.485, *p* < 0.001) at the 3 and 12 months of follow‐up
								Beliefs in the treatment of the illness	Positive Illness Perceptions item treatment control (the individual beliefs in treatment) negatively correlated with anxiety (*ρ* = −0.476 and −0.491, *p* < 0.001) and depressive symptoms (*ρ* = −0.474 and −0.473, *p* < 0.001) at the 3 and 12 months of follow‐up

Of the included studies, three reported psychological factors that were associated with decreased psychological distress (Table 2) [19, 26, 35]. The most common psychological factor that was associated with decreased psychological distress in bladder cancer patients was feeling well informed such that two studies reported a negative correlation between feeling well informed and psychological distress measured by the HADS (depressive: *ρ* = −0.332 and −0.669; anxiety: *ρ* = −0.323 and −0.786 *p* < 0.001), and the FBK‐R23 (*ρ* = −0.335, *p* < 0.001) [26, 35].

### 3.5 Socioeconomic Factors That Increased and Decreased Psychological Distress

Among the included articles, 4 reported socioeconomic factors that were associated with psychological distress (Table 3) [22, 26, 27, 30]. Socioeconomic factors that were associated with increased psychological distress in bladder cancer patients were use of social media (*n* = 1), lower education level (*n* = 1), higher unmet physical functioning/daily living needs (*n* = 1), lower social support (*n* = 1), and high burden of medical expenses (*n* = 1). The socioeconomic factor that was associated with decreased psychological distress in bladder cancer patients was higher social support (*n* = 2) [22, 27]. Heyes et al. reported a negative correlation between higher social support and lower levels of distress (*β* = −0.6, *p* < 0.001) [22].

**TABLE 3:** Associations between socioeconomic factors and psychological distress in bladder cancer patients.

Study	Country	Study design	Primary study objective	Patients (*n*)	Questionnaires	Prevalence (*n*, %)	Setting	Factor	Description of the association
**Studies examining socioeconomic factors that increased psychological distress**									
Mani et al. [26]	Germany	Retrospective cohort	Evaluate the prevalence of mental distress in patients with newly diagnosed bladder cancer	101	HADS and the Fragebogen zur Belastung von Krebskranken (FBK‐R23)	Not reported	Presurgical	Use of social media	Use of social media correlated positively with FBK‐R23 sum scores (*ρ* = 0.240, *p* = 0.018)
Mohamed et al. [27]	United States	Cross‐sectional study	Assess the prevalence of clinical predictors for psychological distress among bladder cancer patients	159	HADS	Not reported	Postsurgical	Lower education level	Psychological distress was significantly associated with lower educational levels (*p* < 0.05)
								Higher unmet physical functioning/daily living needs	Higher psychological distress was associated with higher unmet physical functioning/daily living needs
								Lower social support	Higher distress was associated with lower levels of social support
Qian et al. [30]	China	Prospective cohort	Evaluate the influence of depressive and anxiety symptoms on the 1‐year recurrence rate in nonmuscle invasive bladder cancer patients	104	HADS	Anxiety: 28%, Depression: 33%	Post‐surgical	High burden of medical expense	Heavier burden of medical expenses on the family (odds ratio: 11.46, *p* = 0.001) was associated with higher levels of distress
**Studies examining socioeconomic factors that decreased psychological distress**									
Heyes et al. [22]	Australia	Prospective cohort	Explore the associations between distress and both the functional impact of bladder cancer and its perceived psychological burden	119	The Mini‐mental Adjustment to Cancer	Not reported	Postsurgical	Higher social support	Higher levels of partner support (*β* = −0.6, *p* < 0.001) were associated with lower levels of distress
Mohamed et al. [27]	United States	Cross‐sectional study	Assess the prevalence of clinical predictors for psychological distress among bladder cancer patients	159	HADS	Not reported	Postsurgical	Higher social support	Higher levels of social support were associated with lower levels of distress

## 4 Discussion

With the emotionally taxing nature of bladder cancer and its associated surgical interventions, intravesical and systemic therapies, it has become imperative to delve into their psychological impact on bladder cancer patients. The characterization of factors that have been shown to mitigate psychological distress serves as an important step toward designing interventions that can provide targeted psychological support for bladder cancer patients. Thus, we contribute to these efforts by reporting the prevalence of psychological distress measured by common validated questionnaires such as the HADS and the DT in bladder cancer patients which ranges between 78% and 3.6%. In addition, we show that factors such as younger age, female sex, advanced tumor stage, depressive or anxiety type symptoms, negative perceptions of their cancer and being in the pre‐operative setting comprise the majority of factors that contribute to higher levels of psychological distress in bladder cancer patients. However, factors such as in‐patient rehabilitation, transitioning to the postoperative setting, feeling well informed, and social support can contribute to lower psychological distress in the bladder cancer population. To our knowledge, we present the first comprehensive analysis of the prevalence of and factors affecting psychological distress in bladder cancer patients.

While the broad characterization of predictors and risk factors associated with distress continues to grow in bladder cancer research, studies in other cancer types have shown similar findings to ours [36, 37, 38, 39]. A systematic review reported that patients across cancer types, such as breast and prostate with low social support or advanced cancer stage experienced higher levels of psychological distress in the form of depression and anxiety [37]. A study by Kim et al. showed that female sex was associated with higher psychological distress scores on the Distress Thermometer [38]. In addition, a study in colorectal cancer demonstrated that patients with higher tumor stage reported more psychological distress prior to surgery compared to those with lower tumor stage [36]. Thus, our study expands the literature on distress in oncologic patients by including an examination of factors associated with distress in bladder cancer patients.

Psychological distress in bladder cancer patients has been linked to poorer surgical outcomes [12, 40]. Specifically, one study showed that bladder cancer patients who reported lower scores on the mental health domain of the Short Form 12 prior to radical cystectomy were more likely to experience a severe postoperative complication [40]. Additionally, a study by Chang et al. reported that psychological distress in bladder cancer patients in the form of treatment‐related worry can result in decreased compliance with prescribed therapies and refusal of treatment which can negatively impact cancer‐specific outcomes [41]. Moreover, oncologic patients receiving treatment who experienced higher levels of psychological distress reported decreased quality of communication with their healthcare providers which can similarly negatively impact the delivery of their oncologic care [42]. Given the clinical implications of poorer operative and oncologic outcomes in the setting of elevated psychological distress in bladder cancer patients, the importance of identifying factors that can decrease it cannot be overstated.

Lowering levels of psychological distress in oncologic patients through targeted interventions have been associated with both improved patient‐reported outcomes and cancer‐specific endpoints like survival [43, 44]. Extensive data have linked the development of positive psychological adaptation such as resilience in cancer patients to improved patient‐reported quality of life and lowered stress responses [44]. Moreover, through the mind–body connection, this lowered stress response in patients with cancer has been linked with improved cancer‐specific survival due to decreased activation of the hypothalamic‐pituitary adrenal axis [43]. Thus, it is within this context that interventions to improve the psychological distress and psychological reserve of bladder cancer patients warrant development to improve patient‐specific experiences and clinical care. It stands to reason that a strength of our findings is that they serve as an important springboard for developing strategies geared toward mitigating the disease and treatment‐related psychological distress that bladder cancer patients experience by identifying factors that decrease distress and designing methods that promote them. For example, strategies that enhance a bladder cancer patient's understanding of their illness as well as its associated treatments have the potential to reduce psychological distress and have been employed in other cancer types [45, 46]. A study by Villarreal‐Garza administered a customizable brochure to breast cancer patients to reduce illness uncertainty and in turn reduce the emotional distress related to their complex cancer care [46]. Similarly, strategies and interventions that instilled positive perceptions about their cancer treatment and self‐efficacy in colorectal cancer patients undergoing surgery and systemic therapy improved their distress [47]. Moreover, measures to provide oncologic patients with social support and rehabilitative services have shown clear benefits for enhancing cancer patients' psychological reserve and coping capacity [48, 49]. Thus, these measures can be leveraged by healthcare professionals to reduce the psychological impact of cancer care in bladder cancer patients. Particularly as studies have shown the value of the healthcare team as a key emotional and psychosocial support structure for bladder cancer patients as they navigate their care pathway [50].

### 4.1 Limitations

The limitations of our study include that only articles published in English were included. In addition, the heterogeneity in the study types and designs precluded a formal meta‐analysis and determination of the magnitude of the different associations between given factors and psychological distress. While many studies used similar questionnaires to measure psychological distress, this was not uniform, and some studies used piloted non‐validated instruments which limits some of the comparisons across studies. Moreover, included studies did not exclude patients with clinically diagnosed mental illness which could lead to a poor estimation of psychological distress prevalence rates.

### 4.2 Clinical Implications

Our review suggests that there is a considerable amount of literature focused on factors that are associated with increased distress in bladder cancer patients. Thus, there remains an opportunity for increased study of the factors that decrease psychological distress in this population. The negative impact of high psychological distress on clinical and potentially oncologic outcomes in bladder cancer patients undergoing cancer treatment makes factors that we identified in our review such as feeling well‐informed and social support important targets for the development of strategies designed to improve both bladder cancer survivorship and clinical outcomes.

## 5 Conclusion

Elevated psychological distress in bladder cancer patients has clinical and oncologic implications thus, the identification of factors and the development of strategies that can reduce distress remains of paramount importance. We demonstrate that while clinical factors associated with increased psychological distress are nonmodifiable, factors such as inpatient rehabilitation, feeling informed, and social support can be leveraged by healthcare providers to mitigate most of the distress that bladder cancer patients experience.

## Author Contributions

**Kezia Reji Thomas:** data curation (equal), formal analysis (equal), investigation (equal), writing – original draft (equal), writing – review and editing (equal). **Catherine Joshua:** methodology (equal), resources (equal), writing – review and editing (equal). **Christine Ibilibor:** conceptualization (equal), data curation (equal), formal analysis (equal), funding acquisition (equal), investigation (equal), methodology (equal), supervision (equal), visualization (equal), writing – original draft (equal), writing – review and editing (equal).

## Ethics Statement

The present study does not involve human participants or animal subjects.

## Conflicts of Interest

The authors declare no conflicts of interest.

## Supporting information


Data S1.

## Data Availability

The data that support the findings of this study are available in the Supporting Information of this article.
