# Supplementary material for: Psychological Distress in Bladder Cancer Patients: A Systematic Review
Source: Cancer Med. 2024 Nov 13;13(22):e70345. doi: 10.1002/cam4.70345 (PMC11558900; doi:10.1002/cam4.70345)
Supplement: Supplementary file 1 — Data S1. [file CAM4-13-e70345-s001.docx]

**Supplemental Material/Search Strategies**

| **Database** | **Query** | **Limiters/Expanders** | **Last Run Via** | **Results** |
| --- | --- | --- | --- | --- |
| Pubmed/  MEDLINE | ("bladder cancer*"[tiab] OR "Urinary Bladder Neoplasms"[Mesh] OR "Non-Muscle Invasive Bladder Neoplasms"[Mesh] OR (bladder AND (cancer* OR neoplasm*))) AND ("Psychological Distress"[Mesh] OR "Resilience, Psychological"[Mesh] OR resilience[tiab] OR "mental health"[tiab] OR "mental state"[tiab] OR "Mental Health"[Mesh] OR (("emotional"[tiab] OR "psychological"[tiab]) AND stress[tiab]) OR distress[tiab]) | Limiters –  Pub Dates: 2000-2024  Language: English | Pubmed, including PubmedCentral and MEDLINE  Display mode: Most recent  2/14/2024 | 257 |
| Embase | ('bladder cancer'/exp OR 'bladder cancer' OR 'bladder tumor'/exp OR 'bladder tumor' OR 'bladder neoplasm*')  AND  ('distress syndrome'/exp OR 'distress syndrome' OR 'psychological resilience'/exp OR 'psychological resilience' OR 'emotional stress'/exp OR 'emotional stress' OR 'mental health'/exp OR 'mental health' OR 'psychological distress' OR 'resilience') | Limiters –  Language: English; Population: Adults  Pub Dates: 2000-2024 | Interface – Elsevier  2/14/24 | 404 |
| PsycINFO | DE "Neoplasms" AND DE "Bladder" OR bladder cancer or bladder neoplasms or bladder tumor or urinary neoplasm  OR  DE "Resilience (Psychological)" OR DE "Distress" OR DE "Emotional Health" OR DE "Mental Health" OR psychological distress OR emotion OR resilience | Limiters –  Language: English; Population: Adults  Pub Dates: 2000-2024 | Interface – APA Net  2/15/24 | 70 |
| Web of Science | ("bladder cancer*" OR "Urinary Bladder Neoplasms" OR "Non-Muscle Invasive Bladder Neoplasms" OR (bladder AND (cancer* OR neoplasm*)))  AND  ("Psychological Distress" OR "Resilience, Psychological" OR resilience OR "mental health" OR "mental state" OR "Mental Health" OR (("emotional" OR "psychological") AND stress) OR distress) | Limiters –  Language: English; Document Types: Articles, Review Articles, Correction, Data Paper and Early Access  Pub Dates: 2000-2024 | Clarivate Web of Sciences Core Collection  2/14/24 | 225 |
